# Habituation of Somatosensory Evoked Potentials in Patients with Alzheimer’s Disease and Those with Vascular Dementia

**DOI:** 10.3390/medicina57121364

**Published:** 2021-12-14

**Authors:** Antonio Currà, Lucio Marinelli, Filippo Cotellessa, Laura Mori, Chiara Avanti, Daniela Greco, Manuela Gorini, Paolo Missori, Francesco Fattapposta, Carlo Trompetto

**Affiliations:** 1 Academic Neurology Unit, Department of Medico-Surgical Sciences and Biotechnologies, Sapienza University of Rome, 04019 Terracina, Italy; d.greco@ausl.latina.it (D.G.); manuela.gorini@uniroma1.it (M.G.); 2 Department of Neuroscience, Division of Clinical Neurophysiology, IRCCS Ospedale Policlinico San Martino, 16132 Genova, Italy; lucio.marinelli@unige.it; 3 Department of Neuroscience, Rehabilitation, Ophthalmology, Genetics, Maternal and Child Health, University of Genova, 16132 Genova, Italy; filippo_cotellessa@hotmail.it (F.C.); laura.mori@unige.it (L.M.); chiara.avanti@gmail.com (C.A.); ctrompetto@neurologia.unige.it (C.T.); 4 Department of Neuroscience, Division of Neurorehabilitation, IRCCS Ospedale Policlinico San Martino, 16132 Genova, Italy; 5 Neurosurgery Unit, Policlinico Umberto I, Department of Human Neurosciences, Sapienza University of Rome, 00185 Rome, Italy; paolo.missori@uniroma1.it; 6 Neurology Unit, Policlinico Umberto I, Department of Human Neurosciences, Sapienza University of Rome, 00185 Rome, Italy; francesco.fattapposta@uniroma1.it

**Keywords:** cognitive impairment, somatosensory evoked potentials, habituation, neurophysiology, dementia

## Abstract

*Background and Objectives*: The most prevalent dementia are Alzheimer’s disease and vascular dementia. There is evidence that cortical synaptic function may differ in these two conditions. Habituation of cortical responses to repeated stimuli is a well-preserved phenomenon in a normal brain cortex, related to an underlying mechanism of synaptic efficacy regulation. Lack of habituation represents a marker of synaptic dysfunction. The purpose of this study was to assess the habituation of somatosensory evoked potentials (SEPs) in 29 patients affected by mild-to-moderate Alzheimer’s disease (AD-type) or vascular (VD-type) dementia. *Materials and Methods*: All patients underwent a clinical history interview, neuropsychological evaluation, and neuroimaging examination. SEPs were elicited by electrical stimulation of the right median nerve at the wrist. Six-hundred stimuli were delivered, and cortical responses divided in three blocks of 200. Habituation was calculated by measuring changes of N20 amplitude from block 1 to block 3. SEP variables recorded in patients were compared with those recorded in 15 age- and gender-matched healthy volunteers. *Results*: SEP recordings showed similar N20 amplitudes in AD-type and VD-type patients in block 1, that were higher than those recorded in controls. N20 amplitude decreased from block 1 to block 3 (habituation) in normal subjects and in VD-type patients, whereas in AD-type patients it remained unchanged (lack of habituation). *Conclusions*: The findings suggest that neurophysiologic mechanisms of synaptic efficacy that underneath habituation are impaired in patients with AD-type dementia but not in patients with VD-type dementia. SEPs habituation may contribute to early distinction of Alzheimer’s disease vs. vascular dementia.

## 1. Introduction

Cognitive disorder refers to the abnormality of cognitive function. When abnormality comes from a deterioration that affects multiple cognitive domains, caused by a chronic and progressive process the extent of which overrides that expected from the biological consequences of normal ageing, thereby compromising both professional and social skills, the cognitive disorder is named dementia [1].

The most prevalent dementia is Alzheimer’s disease (AD)—characterized by the progressive accumulation of insoluble deposits of distinct misfolded proteins, the second is vascular dementia (VD) [2]. In AD, the most frequent dementia, amyloid β-protein (Aβ) production by regulated intramembrane proteolysis, is crucial for pathogenicity. An unusual intramembrane cleavage generates various Aβ species, only one of which (the longest variant) is particularly prone to form potentially toxic oligomers. Soluble oligomeric assemblies of amyloidogenic proteins are thought to initiate disease-specific cytopathology and subsequent symptoms. Larger deposits, such as compacted Aβ plaques, seem relatively inert but they might serve as reservoirs of diffusible oligomers [3]. VD refers to a heterogeneous group of cognitive disorders caused by ischemic, hemorrhagic, anoxic, or hypoxic brain damage. Both macrovascular and/or microvascular cerebral disease may induce ischemic cognitive impairment, whereas hypertension is the main cause of hemorrhagic cognitive impairment, by inducing intralobar hemorrhages or multiple petechial hemorrhages [4].

The role of the progressive loss of synaptic proteins in causing dementia received increasing interest since the correlation between synaptic loss and AD was first established [5]. From that pioneering observation, several pre- and post-synaptic proteins have been studied both in AD and VD showing distinct patterns of changes. Data from biochemical studies suggest that not only synaptic molecular structure but also synaptic function may differ between AD and VD [6,7,8]. 

One well-studied mechanism that depends on the integrity of synaptic function is that aimed to autoregulate synaptic activity, i.e., habituation. It consists of a progressive decrement of the response after repeated stimulation. As a sensory stimulus is repeated, the amplitude of the excitatory postsynaptic potential decreases, with no change in membrane properties of postsynaptic cell, indicating that depression of synaptic activity is due to an exclusively presynaptic mechanism (so-called homosynaptic depression) [9]. Depending on how long and frequent the stimulus is, habituation lasts longer (i.e., short-term, that lasts only for a few minutes, or long-term habituation) [10]. 

Many studies demonstrated that habituation may be investigated non-invasively in humans by analyzing the potentials evoked at many levels of the central nervous system by various types of stimuli [11]. A neurophysiological technique ideally suited to investigate how sensory cortices respond to repetitive stimulation consists of testing somatosensory evoked potentials (SEPs). Weak sensory stimuli are repeatedly delivered on peripheral nerves and electrical responses are recorded along the somatosensory pathway. Testing SEPs proved highly sensitive in disclosing abnormal habituation in migraineurs studied interictally, as well as in patients with medication-overuse headache [12]. Investigating SEPs in patients with mild-to-moderate AD or VD dementia would be useful since sensory potentials are generated in a cortical area not directly related to cognitive processes, nor thought to be involved in the early neurodegenerative process of AD [13].

Although great work in differential diagnosis of AD and VD has been performed using evoked potentials [14,15,16], to date, SEPs habituation has not been studied in patients with dementia. We decided to investigate SEPs habituation as a marker of synaptic function in patients with AD-type and VD-type mild-to-moderate dementia. Due to the overlaps in symptomatology, pathophysiology, and risk factors, in their initial phases these two cognitive disorders are not easily distinguished. Having an objective neurophysiological marker may, therefore, assist early recognition of these patients.

## 2. Methods

### 2.1. Subjects

We selected 35 patients affected by mild-to-moderate dementia from the “Centro diagnostico specialistico-Centro territoriale esperto per le demenze Presidio Centro—Distretto 4 AUSL Latina” (Table 1). All participants received a complete description of the investigation and gave their written informed consent to take part in the study. The study was approved by the local ethics committee (Comitato Etico Lazio 2, Protocollo 0182986/2018, date of approval 19 June 2018).

All patients underwent a clinical history interview, a clinical and neuropsychological testing, and neuroimaging examination. Neuropsychological evaluation was based on tests and test battery selected for the specific case from a psychological unstructured interview. The aim of the interview was to reveal traits of anxiety, depression, other psychological distress. After this evaluation, the psychologist selected tests and test batteries tailored for the single patient. Tests included the Mini Mental State Examination, the Rey Auditory Verbal Learning Test with immediate recall and delayed recall, the Recognition Memory Test, the Prose Memory test, the Immediate Visual Recognition Test, the Ideomotor Praxis test, the Oral Praxis Test, Design Copying with and without programming elements, Raven’s Progressive Matrices, the Verbal Span Test forward and backward, the Visual Span Subtest forward, the Visual Span Subtest backward, phonemic, and semantic verbal fluency, the Attentive Matrices Test, the Trail Making test A, B, B-A, the Aachener Aphasie Test, and the Geriatric Depression Scale. Patients exhibiting overt anxiety in the psychological interview, depression or other psychological distress were excluded from the study. All patients evaluated were considered to be affected by mild-to-moderate dementia because they scored 18 to 22 at MMSE (corrected score for age and educational level, Table 1, Table 2 and Table 3). 

According to the NIA-AA [17] and the NINDS-AIREN [18] criteria, 15 patients (age 74.7 ± 6.1 years, 3 men) were diagnosed as having AD (AD-type) and 20 patients (age 80.8 ± 7.1 years, 10 men) as having VD (VD-type). In brief, we considered probable AD patients (AD-type) those exhibiting cognitive decline with insidious onset and no MRI evidence of multiple or extensive infarcts or severe white matter hyperintensity burden. We considered VD patients (VD-type) those exhibiting cognitive deterioration with a sudden onset, step-like progression, and MRI evidence of multiple or extensive infarcts or severe white matter hyperintensity burden.

Patients in the VD-type subgroup that had early SEP component latency longer than that of normal subjects mean value + 2 SD were excluded from the analysis.

SEP variables recorded in patients were compared with those recorded in 15 age- and gender-matched healthy volunteers (age 76.1 ± 6.9; 8 women), having no detectable medical or surgical conditions in place, who documented no current ongoing drug or non-drug treatment for neurological and/or psychiatric disorders, who had MMSE score >28, and reported no trait of anxiety, depression, or other psychological distress at the unstructured psychological interview.

Any participant with a personal history of migraine or taking psychotropic drugs was excluded from the study.

### 2.2. Data Acquisition

SEPs were elicited by electrical stimulation of the right median nerve at the wrist using a constant current square wave pulse (0.1 ms width, cathode proximal), a stimulus intensity set at 1.5 times the motor threshold, and a repetition rate of 4.4 Hz. The active electrodes were placed over the contralateral parietal area (C3′, 2 cm posterior to C3 in the International 10–20 system) and on the fifth cervical spinous process (Cv5), both referenced to Fz; the ground electrode was on the right arm. SEP signals were amplified by Digitimer^TM^ D360 pre-amplifier (Digitimer Ltd., Welwyn Garden City, Hertfordshire, UK) (band-pass 0.05–2500 Hz, gain 1000) and recorded by a CED^TM^ power 1401 device (Cambridge Electronic Design Ltd., Cambridge, UK).

Subjects sat relaxed on a comfortable chair in a well-lit room and were asked to keep their eyes opened and fix their attention on the stimulus-induced thumb movement. During continuous median-nerve stimulation at the wrist, we collected 600 sweeps of 100 ms (post-stimulus), sampled at 5000 Hz. All recordings were averaged off-line using the Signal^TM^ software package version 4.0.2.0.

For the baseline assessment, the 600 artefact-free evoked responses recorded in each participant were averaged (“grand average”). After a digital filtering signal between 0–450 Hz, the various SEP components (N13, N20, P25) were identified according to the latency. Thereafter, we measured the peak-to-peak amplitudes of the cervical N13 component (recorded under the active Cv5 electrode), and the cortical N20 (recorded under the active C3′ scalp electrode and measured as N20-P25 amplitude difference). The 600 evoked responses were partitioned in 3 sequential blocks of 200: responses in each block were averaged off-line (“block averages”), and N20 amplitudes were measured in each block.

### 2.3. Statistical Analysis

We used the Statistical Package for the Social Sciences (SPSS) for all analyses. For the baseline assessment, SEP component amplitudes of the block 1 (trials 1–200) were tested in a one-way analysis of variance (ANOVA) with between-group factor “group” (AD-type vs. VD-type vs. normal subjects, NS). To assess changes in SEP amplitude between blocks in all groups, SEP components amplitudes were tested in a multi-way repeated measures ANOVA with factor “group” and a repeated-measures factor “block” (1 vs. 2 vs. 3). Tukey’s honest significant difference test was used for post hoc analysis.

Pearson’s correlation coefficient was used to test correlations between SEP amplitude in the three blocks and neuropsychological data.

*p* values less than 0.05 were considered to indicate statistical significance.

## 3. Results

Using one-way ANOVA, participants were compared across diagnostic category (controls, AD-type, and VD-type patients). No significant difference was found in demographics (age, years of education) across diagnostic category. In contrast, MMSE scores differed (F_(3,44)_ 4.3, *p* > 0.05). Post hoc analysis showed higher MMSE score in controls than in AD type- and VD-type patients (controls > AD-type = VD-type).

Complete SEP recordings were obtained from all patients and controls participating to the study. None of the participants described the stimulation as painful, nor he/she asked for stopping stimulation during the experimental session. Questioned at the end of the recordings, no subject reported pain.

Six VD-type patients had early SEP cortical component latency longer than + 2 SD the normal value and, therefore, were discarded from the analysis. Thereafter, the VD-type subgroup consisted of 14 patients (age 79.2 ± 6.2 years, six men).

Latency grand averages for all 600 SEP components N13 (NS 12.1 ± 1.3 ms, AD-type 11.9 ± 1.4 ms, VD-type 12.1 ± 1.5 ms, F_(3,44)_, 0.93, *p* > 0.05) and N20 (NS 19.8 ± 1.7 ms, AD-type 19.3 ± 1.6 ms, VD-type 20.1 ± 1.9 ms, F_(3,44)_, 1.03, *p* > 0.05) were similar between groups.

Amplitude grand averages for 600 SEP components N13 (NS 2.3 ± 0.2 µV, AD-type 2.4 ± 0.3 µV, VD-type 2.2 ± 0.4 µV) were similar between groups (F_(3,44)_ = 0.87 *p* > 0.05); amplitude grand averages for 600 SEP components N20 amplitude approximated statistical significance F_(3,44)_ = 1.47, *p* = 0.051; NS 4.7 ± 1.9 µV, AD-type 6.1 ± 1.6 µV, VD-type 5.7 ± 1.7 µV). Post hoc analysis showed higher N20 amplitude in AD type- and VD-type patients than in controls (VD-type > NS, AD-type = VD-type, AD-type > NS).

**Figure 1:**
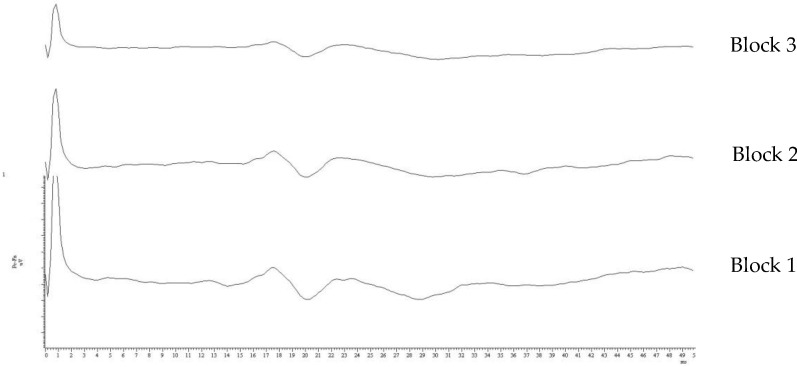
SEPs habituation from block 1 to block 3 in a representative patient with VD-type cognitive disorder. Top row grand average of trials 1-200 (block 1), mid row grand average of trials 201-400 (block 2), bottom row grand average of trials 401-600 (block 3).

**Figure 2:**
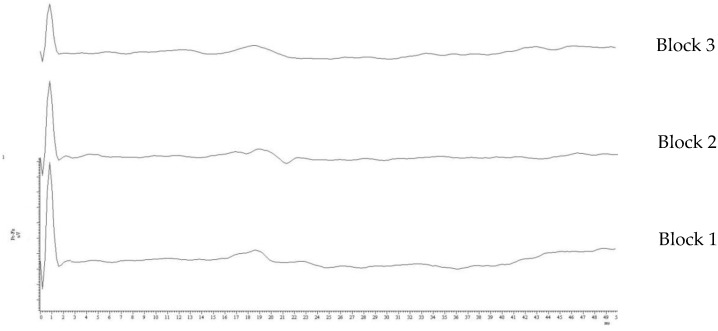
Lack of SEPs habituation from block 1 to block 3 in a representative patient with AD-type cognitive disorder. Top row grand average of trials 1-200 (block 1), mid row grand average of trials 201-400 (block 2), bottom row grand average of trials 401-600 (block 3).

**Figure 3:**
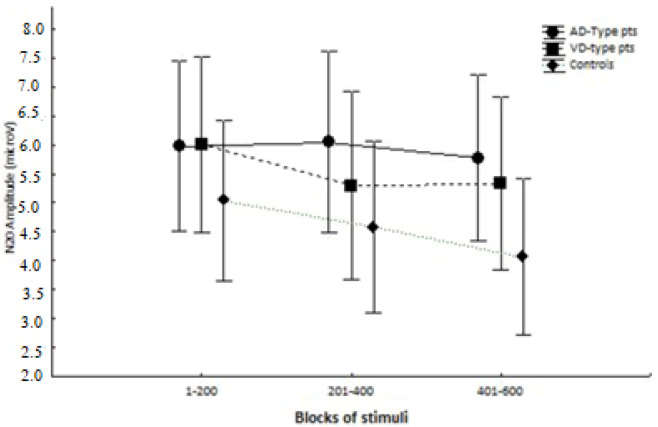
N20-P25 amplitude decreased from block 1 to block 3 in AD-type, VD-type cognitive disorder patients and healthy controls. N20-P25 amplitude remained unchanged from block 1 to block 3 in patients with AD-type cognitive disorder.

ANOVA of N13 amplitude block averages disclosed no main effect (factor “group” (F_(2,44)_ = 0.88, *p* = 0.43) and block (F_(2,44)_ = 0.42, *p* = 0.54). ANOVA of N20 amplitude block averages disclosed a main effect for factors “group” (F_(2,44)_ = 2.98, *p* = 0.034) and block (F_(2,44)_ = 3.12, *p* = 0.03), and a significant interaction of “group” by “block” (F_(5,132)_ = 1.14, *p* = 0.041). Post hoc analysis showed similar N20 amplitudes in AD-type patients and VD-type patients in block 1. N20 amplitude decreased from block 1 to block 3 (i.e., habituation) in normal subjects and in VD-type patients (Figure 1), whereas in AD-type patients (Figure 2) it remained unchanged (lack of habituation) (Figure 3).

Pearson’s test disclosed a correlation between amplitude changes of the N20 component from block 1 to block 3 and MMSE. In AD-type patients, the correlation gave r = 0.32, whereas in VD-type patients it gave r = 0.11.

## 4. Discussion

This study aimed to evaluate the habituation of somatosensory evoked potentials (SEPs) in AD-type and VD-type patients with mild-to-moderate dementia. SEP habituation proved abnormal in AD-type patients, pointing to an impairment of synaptic function in their primary somatosensory cortex, whereas in VD-type patients SEPs habituated normally. By unveiling synaptic dysfunction in a brain region not directly involved in cognitive processes, SEPs habituation makes its way as a neurophysiological marker that helps to distinguish AD-type from VD-type mild-to-moderate dementia.

The N20 component of SEPs showed normal latency in both AD-type and VD-type patients. This finding confirms previous SEPs data in AD [19,20], indicating that cortical neuron loss is not relevant to the occurrence of short latency SEP abnormalities. In VD-type patients, normal N20 latency was conditioned by the recruitment criteria that excluded patients whose vascular damage fell inside the central SEP pathway.

N20 SEP component recorded in block 1 showed greater amplitude in patients than controls. This finding confirms previous studies [21], and it is in line with magnetoencephalography (MEG) evidence showing larger response amplitude of contralateral primary somatosensory cortex in individuals with degenerative mild cognitive impairment than in elderly controls [22]. In VD-type patients, high amplitude of the short latency SEP component may reflect the recruitment of mild-to-moderate patients, because SEP amplitude decrease is reported in severely demented patients [19,23]. Moreover, excluding from the analysis patients having delayed cortical potentials may have favoured the sampling of high amplitude cortical responses.

Increased block 1 N20 amplitude suggests that sensory cortex is more sensitive to stimulation in mild-to-moderate demented patients than in controls. This cortical hyper-sensitivity resembles the phenomenon of cortical sensitization found in headache studies [12]. Sensitization is a phenomenon typically observed in chronic pain [24], a multi-systemic phenomenon regulated by a widely distributed cortical/subcortical network including the contralateral primary somatosensory cortex [25]. Although sensory or pain changes are not typically part of the mild-to-moderate dementia symptomatology, pain has been identified as a risk factor for cognitive dysfunction, which in turn affects pain perception. Recent metanalysis showed that at least one demented patient out of two suffers pain, and that pain prevalence does not differ between dementia subtypes [26]. Furthermore, in demented patients, altered processing of pain and temperature have been described that contributes to differentiate clinical phenotypes [27]. Finally, by measuring the resting-state brain activity before and after selective nerve root block for the treatment of pain in demented patients, changes in MEG neural oscillations in various brain areas were found that represent the transient bridge between pain and cognitive dysfunction [28]. Overall, as a complex experience thought to emerge from the activity of multiple brain areas, pain affects cognitive functions such as attention, memory, and executive function [29], thereby adding value to the present finding of sensory cortex hyper-sensitivity in patients.

Although in AD-type patients N20 latency and amplitude were not impaired, SEPs did not habituate with stimulus repetition. Habituation is a well-preserved phenomenon in the normal brain cortex, probably due to a robust underlying mechanism of synaptic efficacy regulation, therefore lack of habituation represents a marker of synaptic dysfunction. Abnormal SEPs habituation in AD-type patients supports the notion that a cortical pathogenetic mechanism is more relevant than subcortical axonal damage for producing this abnormality. In addition, lack of SEPs habituation marks the synaptic dysfunction in the sensory cortex of patients with AD, a particularly interesting observation, because staging of AD showed that this brain region is involved only at the latest stages of disease (i.e., stage VI) [30]. Conceivably, this discrepancy reflects that dis-habituation is an exquisitely functional abnormality that does not translate into structural alterations visible at histopathological level. Indeed, neuroimaging studies of fractional anisotropy found no difference in the regional pattern of intracortical projecting white matter tracts originating from the somatosensory cortex between AD patients and controls [31]. Although the correspondence between neuropathological staging and clinical staging in AD is not yet well established, early primary somatosensory cortex involvement has been reported in patients with mild cognitive impairment in studies measuring the MEG responses to electric stimuli delivered to median nerves, supporting the idea that the clinical stage of mild cognitive impairment might correspond with stages IV–VI of Braaks’ neuropathological staging of AD [22]. Other investigations also show early sensory cortex dysfunction during AD-type neurodegeneration. Evaluation of EEG coherence between cortical areas showed that the phenotype conversion from mild cognitive impairment to AD is associated with altered connectivity of the sensorimotor cortical network [32]. Resting state EEG showed reduced parieto-occipital alpha activity and increased delta/theta activity in occipital, parietal, and temporal areas in patients with amnesic mild cognitive impairment [33]. Measures of the temporal processing of somatosensory information evaluated by testing the discrimination threshold are abnormal both in patients with mild cognitive impairment and in patients with mild-to-moderate AD [34]. Cortical inhibition tested by short latency afferent inhibition, a technique that explores the inhibition exerted by sensory stimuli on motor areas, is found reduced both in patients with AD [35] and in patients with amnestic mild cognitive impairment [36], thus suggesting that this abnormality begins early in the history of the disease. Paired associative stimulation, a protocol that pairs peripheral sensory and transcranial magnetic motor stimuli for inducing changes in synaptic activity of the sensorimotor pathways like long-term potentiation and long-term depression, induces abnormal responses in patients with mild-to-moderate AD [37]. Overall, several neurophysiological studies in patients with neurodegenerative cognitive disorder up to dementia confirm our finding that in AD the involvement of the sensory cortex takes place earlier than previously thought.

Instead, normal SEP habituation in VD-type patients is not surprising. Since the subcortical areas are mostly perfused by penetrating and deeper branches of the primary feeding arteries, chronic brain perfusion deficit involves mostly the hemispheric subcortical white matter, leaving the cortex intact longer [38]. Considering specifically the sensory cortex, an increased functional network connectivity between default mode and this area has been found to distinguish VD from AD [39]. In support, short-latency afferent inhibition of motor responses evoked by transcranial magnetic stimulation in VD patients is normal [40].

Whereas both AD-type and VD-type patients showed sensory cortex hyper-sensitivity, only AD-type patients combined hyper-sensitivity with hyper-responsiveness (i.e., lack of habituation). This combination reflects a failure of the homeostatic phenomenon described in the “dual process” theory of habituation [41], in which hyper-sensitivity is a facilitatory process that competes with its opposite, habituation, to determine the final outcome after stimulus repetition. In contrast, in VD-type patients this homeostatic mechanism is preserved.

Changes of N20 amplitude from block 1 to block 3 correlated with MMSE scores, and the correlation was significantly stronger in AD-type than VD-type patients. These findings indicate that the SEP amplitude changed less in patients who scored low, thereby suggesting that the synaptic dysfunction increases with increasing cognitive impairment, especially in AD-type dementia. Since the recruited patients were mildly impaired, reasonably low scores represented more widespread (not more pronounced) cognitive deficits. Consequently, the more widespread the deficit is between cognitive domains, the more likely the cortical dysfunction is revealed in cortices not directly involved in cognitive processing.

One limitation of the study is that only VD-type dementia patients having normal SEPs latency were included in the analysis. We considered it necessary to adopt this criterion because we aimed to evaluate the habituation of cortical potentials without confounding factors. Once it has been shown that SEPs habituation can be an objective indicator of the synaptic efficacy, further studies are warranted to understand in detail whether synaptic efficacy may change independently from the integrity of the SEPs neural pathway.

## 5. Conclusions

Investigation of SEPs habituation disclosed synaptic dysfunction in AD but not in VD. Since the differential diagnosis between AD and VD can be challenging [42,43], having a neurophysiological marker can help to distinguish the two forms in the early phase of disease. SEP habituation proved a simple, easy to conduct, often repeatable and inexpensive tool to be used not only for routine diagnosis, but also for research questions to detect dementia in its early preclinical stages, when only few neurons are irreversibly damaged and there is potential for e.g., neuroprotective methods. In addition, testing SEPs habituation at various time points could help in monitoring the disease’s progression, or the effectiveness of potential therapeutic drugs.

## Figures and Tables

**Table 1:** Patients’ demographic data and score on cognitive screening.

Patient	Grp	Age	Sex	Education (Years)	MMSE	Memory Enhancing Drugs
1	AD-type	76	F	5	18	Donepezil 10 mg Memantine 20 mg
2	AD-type	79	M	13	22	None
3	AD-type	62	M	5	20	Galantamine 16 mg
4	AD-type	75	F	8	22	Rivastigmine 9 mg
5	AD-type	68	F	13	21	None
6	AD-type	78	F	0	20	Galantamine 16 mg
7	AD-type	78	F	3	22	Donepezil 5 mg
8	AD-type	80	M	16	21	Memantine 20 mg
9	AD-type	65	F	5	19	None
10	AD-type	68	F	13	22	Rivastigmine patch 4.5 mg
11	AD-type	79	F	5	20	Donepezil 5 mg
12	AD-type	82	F	5	18	None
13	AD-type	74	F	5	21	Galantamine 16 mg
14	AD-type	77	F	5	18	Rivastigmine patch 13.3 mg Memantine 20 mg
15	AD-type	80	F	9	20	Galantamine 16 mg
16	VD-type	86	F	5	18	
17	VD-type	70	F	5	21	
18	VD-type	74	M	5	19	
19	VD-type	75	M	5	22	
20	VD-type	82	M	5	22	
21	VD-type	84	F	5	18	
22	VD-type	85	M	5	20	
23	VD-type	83	F	17	22	
24	VD-type	73	M	8	19	
25	VD-type	80	F	3	21	
26	VD-type	69	F	5	19	
27	VD-type	80	F	4	18	
28	VD-type	89	F	5	18	
29	VD-type	77	M	5	22	

**Table 2:** Neuropsychological tests’ scores in AD-type patients.

		ORIENTATION						MEMORY					ACE-R						FLUENCIES	
Patient	MMSE	Temporal	Spatial	Personal	Raven	Digit for	Digit back	Rey Immediate	Rey Delayed	Prose Immediate	Prose Delayed	Oblivion	Orientation	Memory	Fluency	Language	Visuo-Spatial	Tot.	Phonemic	Semantic
1	18	74	+/-	-									13	8	7	23	9	60		
2	22	96	+	+		4	3	20	0	1	0	1							25	10
3	20	94	+	-						3.3	2	1.3	13	7	5	16	6	47	15	9
4	22	94	+	+	24	4	2			3.3	0	3.3	13	15	6	21	12	67	15	11
5	21	90	+	+/-				18	1										27	7
6	20	87	+	-									12	9	9	21	10	61		
7	22	98	+	+	21	3	2	22	2	5.3	2.3	3	16	16	6	23	13	74	18	9
8	21	88	+	+	28					4.6	1	3.6	14	18	9	25	14	76	23	12
9	19	83	-	+/-	14					4	0	4	9	8	6	17	4	44	11	9
10	22	100	+	+	30	5	4	32	3	5	2.2	2.8	17	11	9	26	15	78	39	11
11	20	90	+/-	-				18	3	5	3	2							1	8
12	18	82	-	-									11	11	8	21	11	62		
13	21	90	+/-	+/-									12	13	6	21	9	61	17	9
14	18	92	+/-	+/-									13	10	7	20	11	61		
15	20	76	+	+	18								11	7	8	17	10	53	24	11
16	18	77	-	-									10	8	6	21	13	58		

**Table 3:** Neuropsychological tests’ scores in VD-type patients.

		ORIENTATION						MEMORY					ACE-R						FLUENCIES	
Patient	MMSE	Temporal	Spatial	Personal	Raven	Digit for	Digit back	Rey Immediate	Rey Delayed	Prose Immediate	Prose Delayed	Oblivion	Orientation	Memory	Fluency	Language	Visuo-Spatial	Tot.	Phonemic	Semantic
17	21	91	+	-	16	4	2	9	1	0	0	0							7	8
18	19	89	+	+/-									12	11	8	22	13	66		
19	22	94	+	+	14					4	0	4	15	11	6	20	9	61	18	10
20	22	100	+	+	20	5	3			3.1	3	1.1	16	13	7	24	14	72	18	9
21	18	88	+	+/-		5	4	17	0	3	0	3							25	13
22	20	100	+	+	17					3	0	3	15	8	8	20	9	60	22	12
23	22	100	+	+	27	5	3	18	0	2.2	2	0	18	11	8	25	14	76	10	12
24	19	86	+	+/-		3	2	19	3	2	2	0							9	5
25	21	91	+/-	+									13	14	5	20	11	63	12	10
26	19	76	+	-									13	10	7	22	10	62		
27	18	78	+/-	+/-									12	10	6	18	12	58		
28	18	75	-	-									12	8	7	22	14	63		
29	22	90	+	+	29					3	0	3	13	10	6	20	13	62	10	13

## Data Availability

The datasets generated during the current study are available from the corresponding author on reasonable request.
